# Utility of the Python package Geoweaver_cwl for improving workflow reusability: an illustration with multidisciplinary use cases

**DOI:** 10.1007/s12145-023-01045-0

**Published:** 2023-07-10

**Authors:** Amruta Kale, Ziheng Sun, Xiaogang Ma

**Affiliations:** 1 Department of Computer Science, University of Idaho, Moscow, ID 83844 USA; 2 Center for Spatial Information Science and Systems, George Mason University, Fairfax, VA 22030 USA; 3 Department of Geography and Geoinformation Science, George Mason University, Fairfax, VA 22030 USA

**Keywords:** Workflow Platforms, Provenance documentation, Common Workflow Language, Reproducibility, Interoperability

## Abstract

Computational workflows are widely used in data analysis, enabling automated tracking of steps and storage of provenance information, leading to innovation and decision-making in the scientific community. However, the growing popularity of workflows has raised concerns about reproducibility and reusability which can hinder collaboration between institutions and users. In order to address these concerns, it is important to standardize workflows or provide tools that offer a framework for describing workflows and enabling computational reusability. One such set of standards that has recently emerged is the Common Workflow Language (CWL), which offers a robust and flexible framework for data analysis tools and workflows. To promote portability, reproducibility, and interoperability of AI/ML workflows, we developed ***geoweaver_cwl***, a Python package that automatically describes AI/ML workflows from a workflow management system (WfMS) named Geoweaver into CWL. In this paper, we test our Python package on multiple use cases from different domains. Our objective is to demonstrate and verify the utility of this package. We make all the code and dataset open online and briefly describe the experimental implementation of the package in this paper, confirming that ***geoweaver_cwl*** can lead to a well-versed AI process while disclosing opportunities for further extensions. The ***geoweaver_cwl*** package is publicly released online at https://pypi.org/project/geoweaver-cwl/0.0.1/ and exemplar results are accessible at: https://github.com/amrutakale08/geoweaver_cwl-usecases.

## Introduction

Scientific workflow management systems (WfMS) like Kepler (Altintas et al. 2004), VisTrails (Callahan et al. 2006), Apache Oozie (Apache Oozie, 2012), Apache Taverna (Apache Taverna, 2014), Apache Airflow (Apache Airflow, 2015), and Geoweaver (Sun et al. 2020) have become increasingly popular. Not only do they support the automation of repetitive tasks, but also capture complex details at various levels and systematically record provenance information for the derived data products (Gil et al. 2007; Kale et al. 2023a). WfMS has emerged as an alternative to ad-hoc approaches for constructing data-intensive machine learning (ML) experiments and provenance tracking. In general, a WfMS can be thought of as a program that consists of a set of modules connected by data flow, where each module can take input data from previous modules, parameter settings, and data from external sources. The visual representation can be considered as a graph, where each node represents the modules and edges represent the flow of data between them. Once the processes are linked together, the WfMS enables users to execute workflows automatically and monitor the progress in real-time.

The growing popularity of workflows has also raised numerous concerns about reproducibility and portability among the scientific community. Ad-hoc methods of data exploration (e.g., Perl scripts) have been widely used in the scientific community but also have significant limitations. This could hamper the collaboration between several researchers unless we standardized computational reusability and portability of workflows. To enable reusability and interoperability, the WfMS communities, for example, the Organization for the Advancement of Structured Information Standard (OASIS) (OASIS, 1998), Workflow Management Coalition (WfMC) (WfMC, 2001), Kepler (Altintas et al. 2004), Galaxy (Goecks et al. 2010), and World Wide Web Consortium (W3C) (Missier et al. 2013) have proposed a series of workflow languages that describe and record these workflow links and the involved processes. The standard languages commonly used in the industrial sector include BPEL (Business Process Execution Language) (Akram et al. 2006), BPMN (Business Process Model and Notation) (Chinosi and Trombetta 2012), and Common Workflow Language (CWL) (Crusoe et al. 2021). For scientific workflows, most WfMS define their own languages, such as Taverna SCUFL2 (Simple Conceptual Unified Flow Language), and YAWL (Yet Another Workflow Language). These workflow languages offer information about the models and describe the process in a portable and reusable manner. Despite the fact that there are numerous WfMS being developed in the community, only a handful of them use the standard languages to describe their workflows. Whereas other WfMS have their unique syntax or approach for describing workflows and infrastructure needs. This approach might restrict computational portability and reusability. As a result, the majority of workflows created cannot be shared among different WfMS. Therefore, choosing the WfMS should be exercised with attention because the process of transitioning the workflow could be complicated and time-consuming especially when qualifying reproducibility. Table 1 highlights the different workflow automation software representing the workflow language and the important features they support.

**Table 1:** Different workflow automation software highlighting the important features

Tool	Process	Workflow Language	Open Source	Features
Kepler	Web services Unix commands Shell script	XML (Extensible Markup Language)	✔	Allows user to reuse data, workflow, and components Freely available under BSD (Berkeley Source Distribution) license
Apache Airflow	Bash Python	DAGs (Directed Acyclical Graphs)	✔	Highly scalable Allows user to monitor and manage task easily
Apache Taverna	Local and remote servers RESTful services Shell script R processor	SCUFL2 (Simple Conceptual Unified Flow Language)	✔	Wide range of services and extensible architecture Workflow provenance Secure
Apache Oozie	Java Hadoop jobs (MapReduce, Pig, Hive, Sqoop) Shell scripts	DAGs (Directed Acyclical Graphs)	***X***	Scalable Reliable Extensible Integrated
VisTrails	Python Local and remote servers Web services	XML (Extensible Markup Language)	✔	Flexible Provenance architecture Support collaborative exploration and visualization
Geoweaver	Python Shell script Local and remote servers Secure Shell	CWL (Common Workflow Language)	✔	Hybrid Workflow Full access to Remote files Process-oriented provenance Code machine separation Hidden data flow

From above Table 1, we have seen different workflow automation software programs and their feature to make scientific workflows reproducible, portable, and provenance-enabled. In particular, in this paper, we would like to draw attention to Geoweaver a WfMS that helps scientists to sort Artificial Intelligence (AI) experiments (create, manage, execute, share, and record) and improves automation and reproducibility of workflows (Sun et al. 2020). Geoweaver is a simple-to-learn and adaptable application that can be used by anyone having prior experience with Python scripting. The accessibility barrier is minimal for reproducing the existing workflows and downloaded workflows can be carried out independently without the need for software installation. Geoweaver has the capability to automate the workflow, record provenance, and export history without worrying about the technical debts and potential loss of their experiment’s history and source code. To ensure interoperability of the designed workflows, we went one step further and automatically translated Geoweaver AI/ML workflows into CWL with our very first Python package ***geoweaver_cwl*** (Kale et al. 2023b). We firmly believe that employing a CWL standard can offer a great solution for describing portable, flexible, and reusable workflows while also reducing the software engineering burden accompanying large-scale data analysis. From another perspective, our work illustrated how an existing workflow platform can further extend its interoperability by enabling the bridge to CWL. Researchers of other workflow platforms can refer to our design and experience if they are planning similar extensions.

Our objective is to highlight the importance of scientific workflows and encourage the research community to adopt the CWL standards. In this paper, we first provide the brief methodology of a provenance-enabled platform named ***Geoweaver*** and the Python package ***geoweaver_cwl*** which transforms Geoweaver AI workflows into CWL. Second, we present several use cases from different domains to test the useability of the derived package. Third, we demonstrate results from the use cases and underline that CWL standards can assist in overcoming major challenges when sharing workflows between institutions and users. Finally, we conclude with some future research directions.

## Methodology of Geoweaver and Geoweaver_cwl

**Fig. 1:**
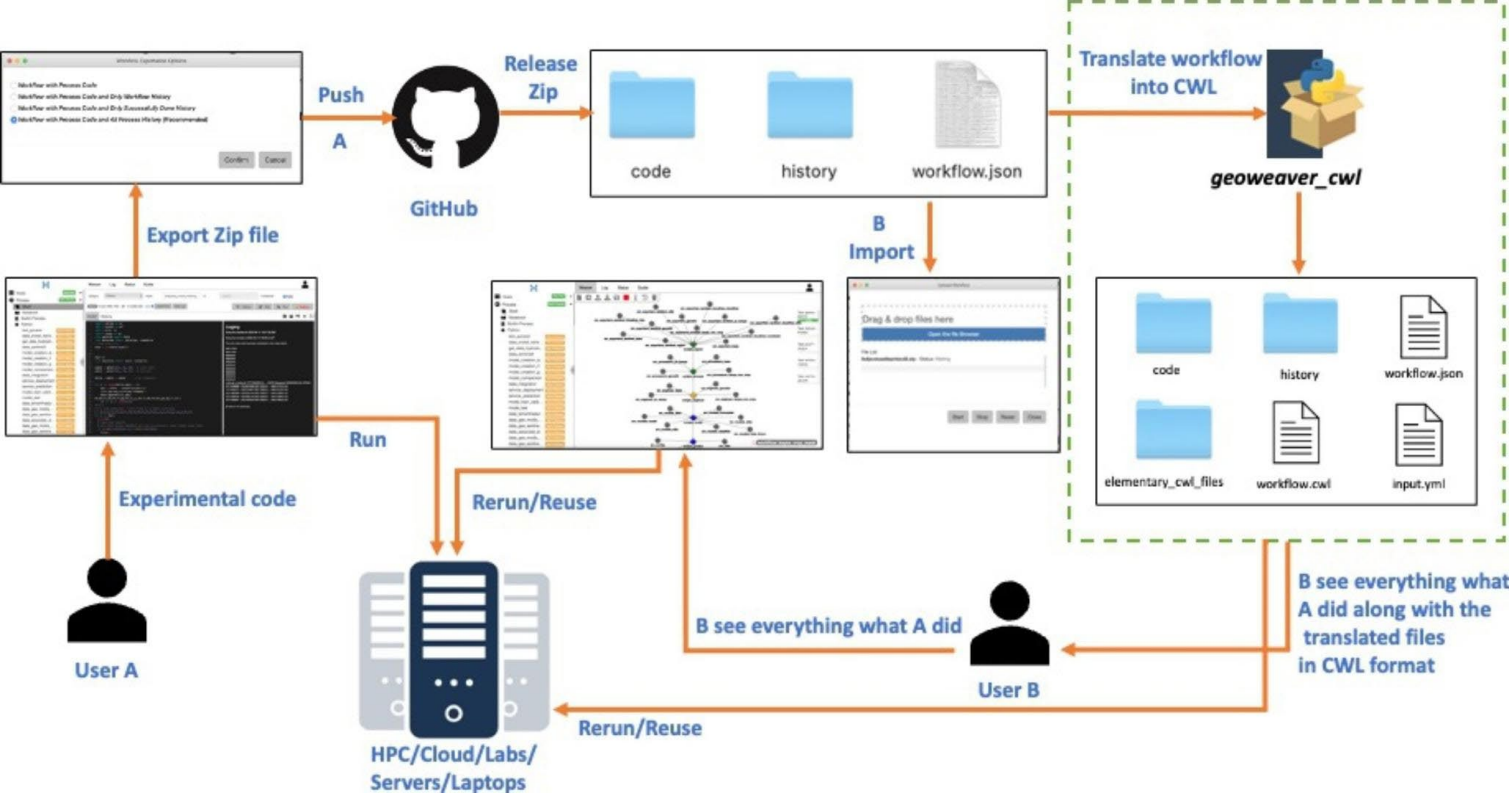
Working structure of Geoweaver and translation of AI/ML workflows shared between user A and user B to enable portability and reproducibility of workflows

Reproducible analyses require sharing data, methodology, and computational algorithms (Peng 2011). In recent years, methods for organizing big data analysis through computational workflow and workflow description language have become increasingly popular to enable reproducibility and interoperability in the earth science domain (Kale and Ma 2023). In this paper, we work on Geoweaver (Sun et al. 2020) as a WfMS tool for researchers and students to improve their research productivity and workflow FAIRness. It is an in-browser software application that allows researchers to create and execute full-stack data processing workflows by utilizing online spatial data resources, high-performance computing environments, and open-source deep learning frameworks. Geoweaver offers a comprehensive solution that includes server management, a code repository, workflow orchestration tools, and a history logger (Sun et al. 2020). We consider Geoweaver as an ideal WfMS which captures crucial provenance data that can reliably trace the history of analytical results. In our previous paper, we demonstrated how to successfully create and execute a workflow in Geoweaver with a simple example (Kale and Ma 2023). One of the major benefits of Geoweaver is that it does not take long to understand, and users can run the Geoweaver workflow package without having Geoweaver installed. Figure 1 demonstrates the workflow shared by user A to user B and how the workflows created in Geoweaver are shareable, reproducible, and standardized in CWL format.

The framework of Geoweaver is based on three core modules host, process, and workflow. The host module is the foundation of the entire framework which opens the entry to existing resources like servers, virtual machines, Jupyter servers, and third-party computing platforms like Google Earth Engine and Google Colab. The process module is widely used to write scripts, programs, commands, or code for the current AI/ML experiments (refer to experimental code in Fig. 1). As most of the AI/ML experiments commonly employ Python, the process module supports Python, Jupyter Notebook, Shell Scripts, and Deep Learning libraries like Keras, TensorFlow, and PyTorch. Provenance support in scientific workflows is paramount, and Geoweaver is ideally positioned to record critical provenance information that can document the lineage of analytical results. The process monitor tracks all the events during the execution and stores all the execution results, inputs, and outputs in the database. The provenance manager is responsible for evaluating the quality of the stored history or fixing the process execution from failures. The workflow module is used to compose workflow by connecting multiple processes into the graphical workflow system. The workflow module also provides real-time status of each executed process indicating a progress bar with a different color. To make the workflow knowledgeable and shareable Geoweaver allows users to import and export (upload and download) the created workflow into a simple zip file with the intent that users can directly start working on the existing files. The zip file contains a code folder, history folder, and workflow.json file. The code folder contains all the experimental code written by a user, the history folder contains the history of all the executed processes in the workflow, and the workflow.json file contains the structured information of nodes and edges that form a workflow. Once the workflow is exported, a zip file can be shared on GitHub or another sharing medium in order for others to reuse the existing work. Any user who wishes to replicate or reproduce the existing work can import the zip file in Geoweaver or can also run the workflow package without having Geoweaver installed.

Despite the fact that workflow systems are popular prior to CWL standards, very few WfMS are compatible with each other. This implies that users who do not follow CWL standards must express their computational workflow differently every time they adopt a new workflow system, resulting in local success but global non-portability. This is due to the lack of standards or practices which makes it difficult for effective collaboration on computational methodologies. To overcome this challenge, we designed ***geoweaver_cwl*** a Python package that automatically describes Geoweaver AI/ML workflows into CWL (Kale et al. 2023b). Describing these workflows into CWL will provide a structured and standard approach when sharing information or reproducing existing work. Once the zip file is exported from GitHub, users can install the ***geoweaver_cwl*** package and easily translate their workflows into CWL scripts. The ***geoweaver_cwl*** has two core functions generate_cwl and generate_yml that enables the easy translations of workflow into CWL files. The Generate_cwl function takes the input workflow.json file and describes the workflow into the workflow.cwl file. Additionally, the function also creates a subdirectory named elementary_cwl_files where all the processes involved in creating a workflow will be translated into CWL files. The generate_yml function produces a Yet Another Markup Language (YAML) file, which writes the input to run the workflow.cwl file. The declarative approach to describe the workflow into CWL scripts keeps provenance organized by documenting the inputs, outputs, workflow steps, and latest version. In addition to Geoweaver, the CWL result can be used on a variety of computer platforms, giving users more opportunities to run the workflow without sacrificing provenance or needing to redo the workflow if they want to use another WfMS. Geoweaver is the unique combination of hybrid workflows, remote access to files, hidden data flow, code-machine separation, well-documented provenance, and standardized CWL-complaint workflows which makes the user experience complete. Using Geoweaver is a long-term investment and will make every scientist’s work preserved and understandable even years after the original experiments.

Although the ***geoweaver_cwl*** package is specifically developed for the Geoweaver platform, researchers of other WfMS can refer to the design and development from it when they build bridges between their systems and the CWL. Such bridges and CWL capability will help establish alignment between the workflow outputs from different WfMS and improve the interoperability. Our previous paper (Kale et al. 2023b) gives a detailed description about the design, structure, and functionality of modules in the ***geoweaver_cwl*** package. This paper will illustrate the utility of the package through a variety of use cases listed below.

## Use cases, results, and evaluation

A comprehensive explanation of the technical framework of ***geoweaver_cwl***, specifically the functions “generate_cwl” and “generate_yml,“ has been provided in our previous open access paper (Kale et al. 2023b). The detailed description elaborates on the creation and implementation of these functions within the ***geoweaver_cwl*** package. We have previously tested ***geoweaver_cwl*** with simple and complex workflows provided by Geoweaver. However, to verify the utility of our package, we decided to validate with more use cases from different domains. Below is a list of five different use cases we have tested on our package.

### Use case 1: CMAQ-predict-geoweaver

The scientific topic of this workflow is to monitor and predict the air quality index in California, that integrates the conventional air quality model, the Community Multi-scale Air Quality Model (CMAQ), and AI models. This workflow was created by Geoweaver and is publicly available on GitHub (https://github.com/earth-artificial-intelligence/cmaq-predict-geoweaver). The GitHub repository contains the code folder, history folder, and workflow.json file.

We installed the ***geoweaver_cwl*** package and used the generate_cwl and generate_yml functions to translate this workflow into CWL. We obtained the files “input.yml”, “workflow.cwl”, and “elementary cwl files folder”, which included the cwl files used in creating the workflow. The workflow translation process was fast and easy, and we also noticed that using cwltool speeds up workflow execution compared to the original procedure in Geoweaver. The translated code for this use case is also made available on GitHub: https://github.com/amrutakale08/geoweaver_cwl-usecases/tree/main/cmaq-predict-geoweaver-master.

**Fig. 2:**
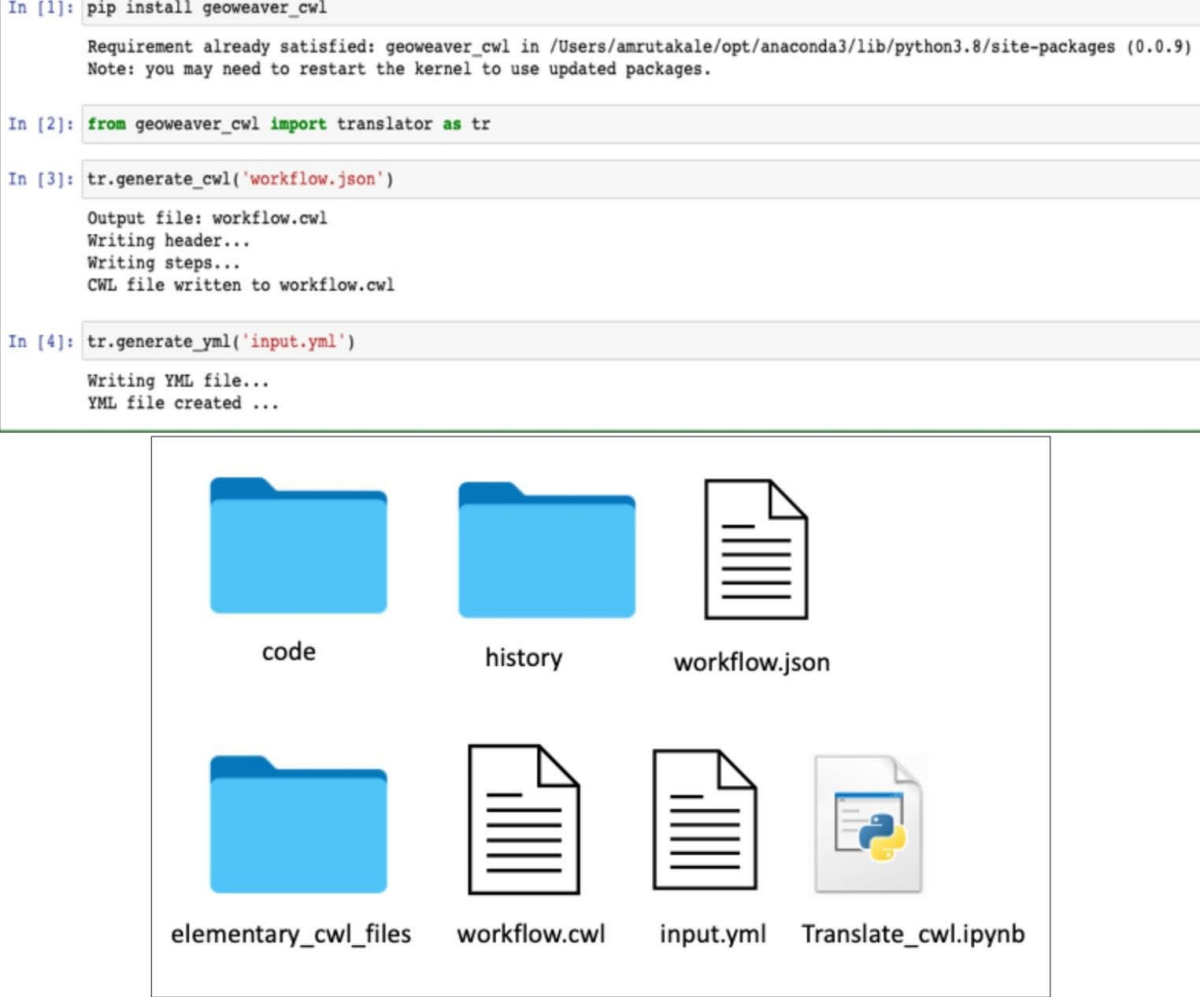
Installation of ***geoweaver_cwl*** package with the functions generate_cwl and generate_yml to successfully translate the Geoweaver workflows into CWL (top). Translated CWL files which consist of elementary_cwl_files folder, workflow.cwl, input.yml, and the translate_cwl.ipynb file (Python file where we have written the code to install the package and try to run functions generate_cwl and generate_yml) (bottom)

Once the workflows are written into CWL scripts they can be executed on a variety of software environments like cwltool, Arvados, Toil, CWL-Airflow, and more. Additionally, there are several cutting-edge applications like Rabix that can speed up the procedure. Rabix is an open-source desktop application that allows researchers to create and edit CWL documents (Kaushik et al. 2017). In our previous paper (Kale et al. 2023b), we used cwltool to execute the CWL scripts generated from the Python package. We invoke cwl_runner with workflow.cwl and input object input.yml on the command line.


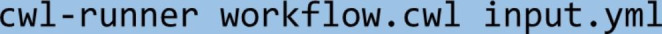


The command is intended to provide the same outcomes as workflow files in Geoweaver by triggering all the CWL and YAML files’ internal functions in the same order. The benefit of CWL offers a way to describe reusable workflows. Researchers and students from any domain can easily share, exchange, edit, and reuse workflows by translating Geoweaver AI/ML workflows into CWL scripts using the ***geoweaver_cwl*** package. Another advantage of CWL is that applications written with CWL are portable and can be used in a multitude of environments, such as local or cloud infrastructures.

### Use case 2: Emission-AI-geoweaver

This workflow was generated by Geoweaver to replicate the experiments done in Emission AI’s published article, which is to build ML models to train on satellite observations (Sentinel 5), ground observed data (EPA eGRID), and meteorological observations (MERRA) data to directly predict the Nitrogen Dioxide (NO2) emission rate of coal-fired power plants. This workflow is publicly available on GitHub (https://github.com/earth-artificial-intelligence/emissionai-geoweaver) with all the necessary files. We followed similar steps as Fig. 2 (left) to translate the workflow in CWL and successfully generated CWL files. The translated workflow code and files are available on GitHub: https://github.com/amrutakale08/geoweaver_cwl-usecases/tree/main/emissionai-geoweaver-main.

### Use case 3: Eddy-detection-geoweaver

This workflow was generated by Geoweaver to replicate the experiment from the Jet Propulsion Laboratory (JPL) notebook. The workflow aims to train an ML model and use it to detect ocean eddies from remote sensing imagery automatically. This workflow is available on GitHub (https://github.com/earth-artificial-intelligence/eddy_detection_geoweaver). Similar to the above two use cases, the translation to CWL was a fluent process. The translated CWL workflow code and files are available at: https://github.com/amrutakale08/geoweaver_cwl-usecases/tree/main/eddy_detection_geoweaver-main.

### Use cases 4 and 5: Interdisciplinary use cases

The majority of the use cases we used in this paper and our previous paper (Kale et al. 2023b) were acquired from Geoweaver. To confirm the portability and reliability of our package we tested a few other use cases from different domains. One demonstrates the proposed framework of ML application in determining Multiple Sclerosis (MS) types and progression levels in MS patients. The other demonstrates the data preprocessing steps for the Moderate Resolution Imaging Spectroradiometer (MODIS) satellite data. Although these two workflows are much simpler and smaller than the above use cases (1, 2, and 3), the results make it certain that ***geoweaver_cwl*** is able to handle the workflows in different research domains. The workflows created in Geoweaver are publicly available on GitHub: https://github.com/amrutakale08/workflows. The translated CWL workflows are available at: https://github.com/amrutakale08/geoweaver_cwl-usecases/tree/main/MS-patients and https://github.com/amrutakale08/geoweaver_cwl-usecases/tree/main/ModisDataProcess.

## Conclusions and future work

The issue of standardizing computational workflows is becoming increasingly significant and has a prominent impact on the research community. Various sectors in science, industry, and government have already transitioned to workflows, but the initiatives focusing on portability, scalability, and standardization are still limited and fragmented. In this paper, we call attention to this issue and provide a community-driven solution ***geoweaver_cwl***, which addresses the current struggles in attaining portability, reproducibility, and scalability of workflows. We evaluated ***geoweaver_cwl*** using various use cases from different domains. The study indicates that the ***geoweaver_ cwl*** package can greatly assist researchers of the different domains in translating their AI/ML workflows into CWL-compliant WfMS software applications. As the project progresses and expands, we plan to incorporate additional use cases from diverse domains, which will facilitate further quantification and validation, such as running time, scalability, and provenance documentation, of the framework in future studies. Although most of our use cases are in the field of geoscience, we have also tested use cases in other domains and proven the utility of our package. In future work, we will collect feedback from users and update the package by adding new functionality. The current package offers basic workflow translation of Geoweaver workflows into CWL scripts. In the future, we want to enhance it by including more detailed workflow steps in order to provide more insight to the user. We encourage the research community to embrace WfMS and adopt the CWL standards in creating and sharing portable and complete workflow descriptions.

## Data Availability

The ***geoweaver_cwl*** Python package is made open access at: https://pypi.org/project/geoweaver-cwl/0.0.1/. The source code of the package is accessible at: https://github.com/amrutakale08/geoweaver_cwl and exemplar use cases are accessible at: https://github.com/amrutakale08/geoweaver_cwl-usecases. The source code of the Geoweaver platform is accessible at: https://github.com/ESIPFed/Geoweaver. Additionally, readers can reach Xiaogang Ma for more information: max@uidaho.edu.
